# Identification of the SRC-family tyrosine kinase HCK as a therapeutic target in mantle cell lymphoma

**DOI:** 10.1038/s41375-020-0934-6

**Published:** 2020-06-26

**Authors:** Hildo C. Lantermans, Marthe Minderman, Annemieke Kuil, Marie-José Kersten, Steven T. Pals, Marcel Spaargaren

**Affiliations:** 1 Department of Pathology, Cancer Center Amsterdam, Amsterdam UMC, University of Amsterdam, Amsterdam, The Netherlands; 2 Lymphoma and Myeloma Center Amsterdam – LYMMCARE, Amsterdam, The Netherlands; 3 Department of Hematology, Cancer Center Amsterdam, Amsterdam UMC, University of Amsterdam, Amsterdam, The Netherlands

**Keywords:** B-cell lymphoma, Targeted therapies, Oncogenes

## Abstract

Mantle cell lymphoma (MCL) is an aggressive non-Hodgkin lymphoma subtype arising from naïve B cells. Although novel therapeutics have improved patient prognosis, drug resistance remains a key problem. Here, we show that the SRC-family tyrosine kinase hematopoietic cell kinase (HCK), which is primarily expressed in the hematopoietic lineage but not in mature B cells, is aberrantly expressed in MCL, and that high expression of HCK is associated with inferior prognosis of MCL patients. HCK expression is controlled by the toll-like receptor (TLR) adaptor protein MYD88 and can be enhanced by TLR agonists in MCL cell lines and primary MCL. In line with this, primary MCL with high HCK expression are enriched for a TLR-signaling pathway gene set. Silencing of HCK expression results in cell cycle arrest and apoptosis. Furthermore, HCK controls integrin-mediated adhesion of MCL cells to extracellular matrix and stromal cells. Taken together, our data indicate that TLR/MYD88-controlled aberrant expression of HCK plays a critical role in MCL proliferation and survival as well as in retention of the malignant cells in the growth- and survival-supporting lymphoid organ microenvironment, thereby contributing to lymphomagenesis. These novel insights provide a strong rationale for therapeutic targeting of HCK in MCL.

## Introduction

Mantle cell lymphoma (MCL) is an aggressive lymphoma subtype with poor clinical outcome, characterized by the t(11;14)(q13;q32) translocation, resulting in overexpression of Cyclin D1. Advances in MCL therapy have improved patient prognosis, but due to primary and secondary resistance there is a high clinical need for novel therapeutic targets [1].

The SRC-family tyrosine kinase hematopoietic cell kinase (HCK), which is predominantly expressed in the hematopoietic lineage, has been implicated in various cellular processes including chemokine signaling, proliferation, apoptosis, and immune cell activation [2–5]. Furthermore, HCK has been shown to be deregulated in hematological and solid malignancies, and plays a role in tumor cell survival [6–8]. In Waldenström’s Macroglobulinemia (WM) and activated B-cell type diffuse large B-cell lymphoma (ABC-DLBCL) cells harboring the pathogenic MYD88-L265P mutation, mutant MYD88-dependent HCK expression was observed and HCK knockdown reduces cell viability, implying that HCK targeting could be beneficial in patients with lymphomas with mutant MYD88 [8]. Although no MYD88 mutations have been reported in MCL, MYD88-regulatory toll-like receptors (TLRs) are upregulated and TLR stimulation supports cell proliferation and survival [9–11]. Here, we show that HCK is aberrantly expressed in MCL in a TLR- and MYD88-dependent fashion and that high HCK expression correlates with poor prognosis. Furthermore, HCK controls proliferation and survival as well as integrin-mediated adhesion of MCL cells. Our results indicate that HCK inhibition has therapeutic potential in MCL, and possibly also in other lymphomas that do not harbor MYD88 mutations.

## Methods

For information about shRNA cloning, cell-culture, transductions, stimulations, immunoblotting, primary cell isolation, RT-qPCR, cell-cycle analysis, Annexin-V staining, integrin staining, and Gene Set Enrichment Analysis (GSEA) see Supplementary “Materials and Methods”.

For adhesion experiments, JeKo-1 cells expressing inducible shRNA’s were pre-treated with doxycycline and allowed to adhere to 96-well-plates, coated with fibronectin or the stromal cell line HS-27a expressing GFP, in the absence/presence of phorbol-12-myristate 13-acetate (PMA). To quantify adhesion to HS-27a, cells were trypsinized and quantified by flow cytometric analysis. As indicated, JeKo-1 or primary MCL cells were treated for 30 min at 37 °C (A419259) or 4 °C (HP2/1, TS1/22) prior to allowing cells to adhere to fibronectin-coated plates or the stromal cell line HS-27a-GFP, For further details see Supplementary “Materials and Methods”.

## Results and discussion

**Fig. 1:**
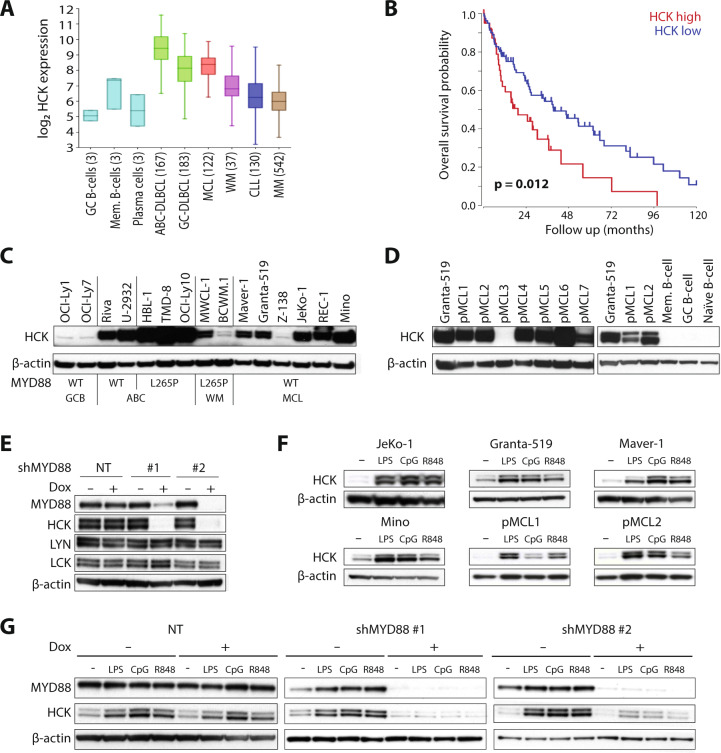
Aberrant HCK expression in MCL is associated with poor patient survival and controlled by TLR/MYD88-signaling. **a** *HCK* mRNA expression in MCL patients. Publicly available micro-array datasets of normal B-cell subsets and various B-cell malignancies were analyzed for *HCK* expression. In MCL, *HCK* mRNA is upregulated in comparison to normal B-cell subsets, WM, CLL, and MM. *HCK* expression levels in MCL are similar to those in GC-DLBCL. Between brackets the number of patients per dataset. **b** High *HCK* mRNA expression is correlated with poor MCL patient prognosis. The GSE93291 micro-array dataset containing 122 MCL patients was used to evaluate the prognostic value of *HCK* expression for the tertile of patients with the highest HCK expression (HCK high, *n* = 41) versus the rest (HCK low, *n* = 81). Kaplan-Meier curves for overall survival probability are shown, *p* = 0.012 by the log-rank test. **c** HCK protein expression across a panel of various B-cell malignancies. Immunoblot, β-actin was used as a loading control. MYD88 status indicates whether MYD88 is wildtype (WT) or mutated in each cell line. GCB = GC-DLBCL, ABC = ABC-DLBCL (**d**) HCK protein expression in primary MCLs and normal B-cells subsets. Immunoblot, β-actin was used as a loading control. **e** HCK, LYN, and LCK protein expression of JeKo-1 cells transduced with pLKO-TET-puro plasmids encoding two shRNA’s targeting MYD88 or a scrambled shRNA (NT). Cells were treated with doxycycline (dox) for 7 days. Immunoblot, β-actin was used as a loading control. **f** HCK protein expression in MCL cell lines and primary patient cells stimulated with TLR-ligands LPS, CpG, or R848 for 48 h. Immunoblot, β-actin was used as a loading control. **g** HCK protein expression in JeKo-1 cells expressing two shRNAs targeting MYD88 or a scrambled shRNA (NT). Cells were treated with doxycycline (dox) for 3 days, followed by stimulation for 48 h with LPS, CpG, or R848 in the absence or presence of doxycycline. Immunoblot, β-actin was used as a loading control.

To evaluate *HCK* mRNA expression across various lymphoid malignancies, we analyzed publicly available microarray data. In germinal center B-cells and normal plasma cells *HCK* was below the detection-threshold, whereas it was weakly expressed in memory B-cells (Fig. 1a). In tumor biopsies of ABC- and Germinal Center (GC)-DLBCL *HCK* was expressed, with higher levels of HCK in ABC-DLBCL (Fig. 1a). Expression of *HCK* in MCL was comparable to GC-DLBCL and higher than in WM, multiple myeloma (MM), or chronic lymphocytic leukemia samples (Fig. 1a). Importantly, high *HCK* expression was found to correlate with poor overall survival in MCL patients. The median overall survival was 17 months in HCK-high vs. 40 months in HCK-low patients and the 5 year survival was 14% in HCK-high vs. 41% in HCK-low patients (Fig. 1b).

Next, we analyzed HCK protein expression in a panel of WM, DLBCL and MCL cell lines. In the MCL cell lines Maver-1, Granta-519, JeKo-1, Rec-1, and Mino HCK levels were similar to ABC-DLBCLs without MYD88 mutations, whereas in Z-138 HCK was barely detectable (Fig. 1c). Notably, Z-138 represents an atypical MCL cell line, with blastoid transformation in the terminal phase of disease. In GC-DLBCL cell lines HCK was hardly detectable, whereas it was expressed in all ABC-DLBCL cell lines independent of the presence of a MYD88 mutation, although the levels were higher when MYD88 was mutated (Fig. 1c). Notably, the WM cell lines, expressing mutant MYD88, contained less HCK than most MCL lines (Fig. 1c). Higher expression of HCK was observed in the WM cell line MWCL-1 than in BCWM.1, in accordance with a previous study [8]. In addition, we also analyzed HCK expression in primary MCLs alongside healthy B-cell subsets. Whereas HCK mRNA and protein were hardly detectable in naïve, GC and memory B-cells, or in plasmablasts (Fig. 1d & Supplementary Fig. 1), high expression of HCK mRNA (Fig. 1a, Supplementary Fig. 1) and protein (Fig. 1d) was observed in 6 out of 7 primary MCLs, at similar levels as in Granta-519 (Fig. 1d). Taken together, HCK is aberrantly upregulated in MCL, high HCK expression correlates with poor patient survival, and enhanced HCK expression in B-cell malignancies is not dependent upon oncogenic MYD88 mutations.

To investigate whether HCK expression depends on (wild type) MYD88, we transduced JeKo-1 with doxycycline-inducible MYD88 shRNAs. MYD88 knockdown strongly reduced HCK protein and RNA levels, indicating that MYD88-mediated signaling drives HCK transcription in JeKo-1 (Fig. 1e, Supplementary Fig. 2). In contrast, underlining the specificity of this effect, expression of the Src family kinases Lyn, Lck, and Fyn was not affected (Fig. 1e and data not shown). Notably, HCK expression was not reduced upon serum deprivation of the cells, indicating a cell intrinsic mechanism for MYD88-dependent HCK expression (data not shown). Since MYD88 plays a prominent role in TLR-signaling and TLR agonists from necrotic cells are abundantly present in the tumor microenvironment, we stimulated MCL cells with TLR agonists and analyzed HCK levels. Stimulation of JeKo-1, Granta-519, Maver-1, Mino, and primary MCL with TLR agonists enhanced *HCK* mRNA (Supplementary Fig. 3) and HCK protein levels (Fig. 1f), in a MYD88-dependent manner (Fig. 1g). Hence, HCK expression in MCL depends on constitutive MYD88 signaling and can be further enhanced by TLR stimulation. In line with these findings, GSEA of MCL patients with high versus low HCK expression showed enrichment of the KEGG TLR signaling pathway gene set (Supplementary Table 1 and Supplementary Fig. 4).

**Fig. 2:**
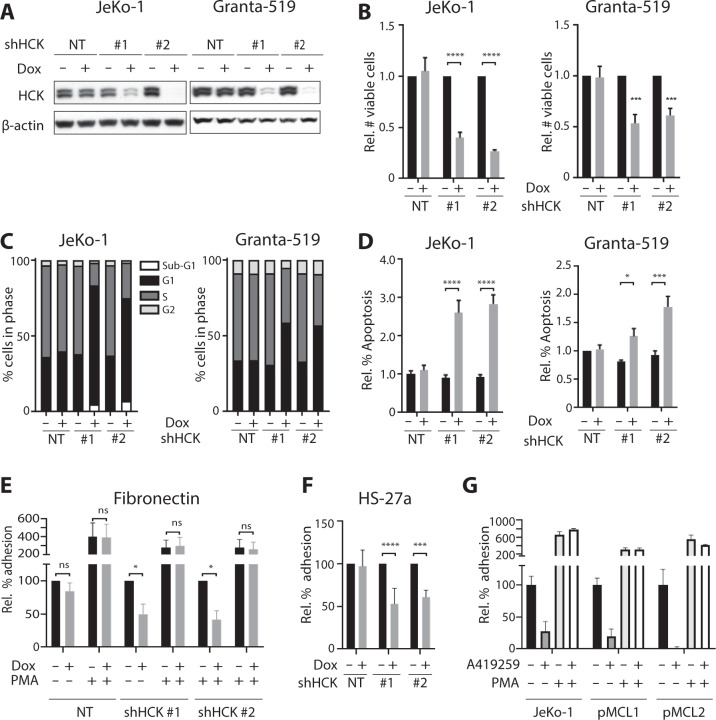
HCK is critical for proliferation, survival and integrin-mediated adhesion of MCL cells. **a**–**f** JeKo-1 and Granta-519 cells were transduced with pLKO-TET-puro plasmids encoding two shRNA’s targeting HCK or a scrambled shRNA (NT). **a** HCK protein expression in MCL cell lines after 3 days of doxycycline treatment. Immunoblot, β-actin was used as a loading control. **b** Number of viable cells after HCK knockdown, determined by flow cytometric analysis and 7-AAD staining after 7 days of doxycycline treatment. Number of viable cells was normalized to the untreated condition. Data are presented as mean ±   S.E.M. of four independent experiments performed in triplicate. **c** Cell cycle analysis after HCK knockdown. The percentage of cells in Sub-G1 (BrdU^-^, <To-Pro-3^-^), G1 (BrdU^-^,To-Pro-3^-^), S (BrdU^+^), and G2 (BrdU^+^, To-Pro-3^+^) were determined by flow cytometric analysis after 7 days of doxycycline treatment. The graphs are representative for 3 individual experiments. **d** Apoptosis after HCK knockdown, defined as the percentage of Annexin-V positive cells after 7 days of doxycycline treatment. Percentage of Annexin-V positive cells were normalized to the untreated condition. Data are presented as mean ±  S.E.M. of three independent experiments. **e** Integrin-mediated adhesion of JeKo-1 cells after HCK knockdown. Cells were treated with doxycycline for 4 days and allowed to adhere to fibronectin-coated plates for 30 min in the presence or absence of PMA. Non-adherent cells were removed by extensive washing, and adherent cells were quantified. Percentage of adherent cells were normalized to the untreated condition. Data are presented as mean ±  S.E.M. of two independent experiments performed in triplicate. **f** Stromal cell adhesion of JeKo-1 cells after HCK knockdown. Jeko-1 cells were treated with doxycycline for 5 days and allowed to adhere to a monolayer of HS-27a-GFP stromal cells for 30 min. Non-adherent cells were removed by extensive washing, and adherent cells were quantified by flow cytometric analysis to separate HS-27a-GFP from JeKo-1 cells. Percentage of adherent cells were normalized to the untreated condition. Data are presented as mean ±  S.E.M. of four independent experiments performed in triplicate. **g** Integrin-mediated adhesion of primary MCL or JeKo-1 cells treated for 30 min with 100 nM A419259 and allowed to adhere to fibronectin-coated plates for 30 min. Non-adherent cells were removed by extensive washing, and adherent cells were quantified. Percentage of adherent cells were normalized to the untreated condition. *P < 0.05; **P < 0.01; ***P < 0.001; ****P < 0.0001 using two-way ANOVA with Sidak multiple comparison test.

Since HCK has been identified as a pro-survival kinase in MYD88-mutated ABC-DLBCL and WM cell lines [8], we investigated if HCK is involved in MCL cell survival. JeKo-1 and Granta-519 were transduced with doxycycline-inducible HCK shRNAs and in both cell lines knockdown of HCK resulted in a significant decrease in the number of viable cells (Fig. 2a, b). To discriminate between effects on cell proliferation and apoptosis, we performed BrdU cell proliferation and Annexin-V apoptosis assays following HCK knockdown. In both cell lines HCK knockdown increased the percentage of cells in G1 and decreased the percentage of cells in S-phase (Fig. 2c). Furthermore, an increase in the amount of Annexin-V positive cells was observed after HCK knockdown (Fig. 2d). These data demonstrate that HCK regulates proliferation, i.e., G1/S transition, and survival of MCL cells.

As illustrated by the molecular mechanism underlying the clinical efficacy of the BTK inhibitor ibrutinib in MCL patients, MCL cells are critically dependent upon the interaction with their tumor microenvironment for in vivo survival and growth, and integrin-mediated adhesion is crucial for retention of the MCL cells in their protective lymphoid organ niche [12–14]. Interestingly, the integrin-mediated adhesion of MCL cells to fibronectin and the bone marrow stromal cell line HS-27a was significantly impaired after HCK knockdown (Fig. 2e, f). Notably, integrin activation by stimulation of PKC with PMA rescued this effect (Fig. 2e), demonstrating that the impaired adhesion upon HCK silencing does not reflect reduced cell viability or integrin expression (Supplementary Fig. 5a) and that PKC controls integrin activity independent or downstream of HCK. Pretreatment of MCL cells with the integrin α4 blocking antibody HP2/1 completely abrogated the adhesion to fibronectin and diminished the adhesion to the bone marrow stromal cell line HS-27a by more than 85% (Supplementary Fig. 5b), demonstrating the involvement of integrin α4β1, also in the interaction with stromal cells. Finally, to assess a possible role for HCK in adhesion of primary MCL, we employed a potent inhibitor of HCK, the pan-Src family kinase inhibitor A419259 [15]. Both primary MCL tested, as well as JeKo-1 cells, showed a strong dose-dependent reduction in the adhesion to fibronectin upon treatment with A419259 (Fig. 2g & Supplementary Fig. 5c), and stimulation of PKC with PMA rescued this effect (Supplementary Fig. 5c). Although inhibition of other SFKs may also contribute to the observed reduction in adhesion, these data support a potential involvement of HCK in regulation of integrin-mediated adhesion of primary MCL cells as well.

Taken together, we demonstrate that HCK is aberrantly upregulated in MCL cell lines and primary patient samples, and high expression of HCK correlates with inferior prognosis. In MCL, HCK expression is MYD88-dependent, but not driven by mutant MYD88. Furthermore, TLR/MYD88-signaling transcriptionally regulates HCK levels. Thus, the aberrant HCK expression in primary MCL cells may reflect local TLR stimulation in the tumor microenvironment. This notion is supported by the GSEA, showing enrichment of a TLR signaling pathway gene set in MCL patients with high expression of HCK. Functionally, HCK knockdown results in G1/S arrest and impaired cell viability. In addition, we established that HCK regulates integrin-mediated adhesion of MCL cells to the extracellular matrix and stromal cells. By analogy to our studies on the mechanism of action underlying the clinical efficacy of ibrutinib [12–14], this implies that HCK inhibition could mobilize MCL cells from their protective lymphoid organ niche into the circulation, thereby depriving them from critical growth- and survival-factors. Combined with the restricted expression of HCK in hematopoietic cells, enhancing the likelihood of clinical safety, our results provide a strong rationale for clinical studies exploring the efficacy of targeted therapy with HCK inhibitors in MCL.

## Supplementary information

Supplemental Figure 1-3

Supplemental Figure 4

Supplemental Figure 5

Supplemental Table 1

Supplemental Materials and Methods
