# Supplementary material for: Identification of the SRC-family tyrosine kinase HCK as a therapeutic target in mantle cell lymphoma
Source: Leukemia. 2020 Jun 26;35(3):881–6. doi: 10.1038/s41375-020-0934-6 (PMC7932922; doi:10.1038/s41375-020-0934-6)
Supplement: Supplementary file 1 — Supplemental Figure 1-3 [file 41375_2020_934_MOESM1_ESM.pdf]

Supplementary Figures, Lantermans et al.

Supplementary Figure 1

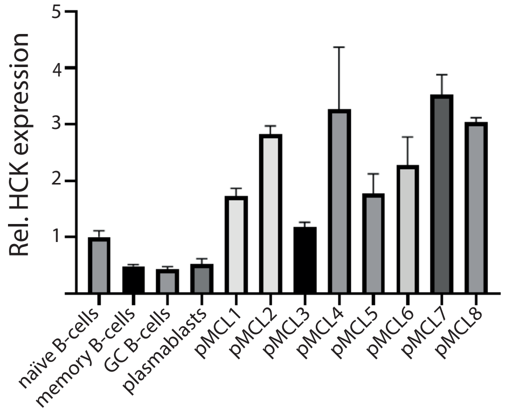

HCK mRNA expression in normal B-cells or primary MCL material determined by RT-qPCR. RPLP0 was used as an input control. Data were normalized to the value of naïve B-cells

Supplementary Figure 2

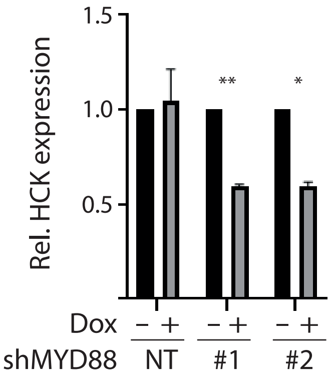

HCK mRNA expression after MYD88 knockdown determined by RT-qPCR. Jeko-1 cells expressing two doxycycline-inducible shRNA targeting MYD88 or a non-targeting shRNA (NT) were treated for 7 days with doxycycline. Data were normalized to the value of untreated cells. RPLP0 was used as an input control. Data are presented as mean +/- S.E.M of three independent experiments performed in triplicate.

Supplementary Figure 3

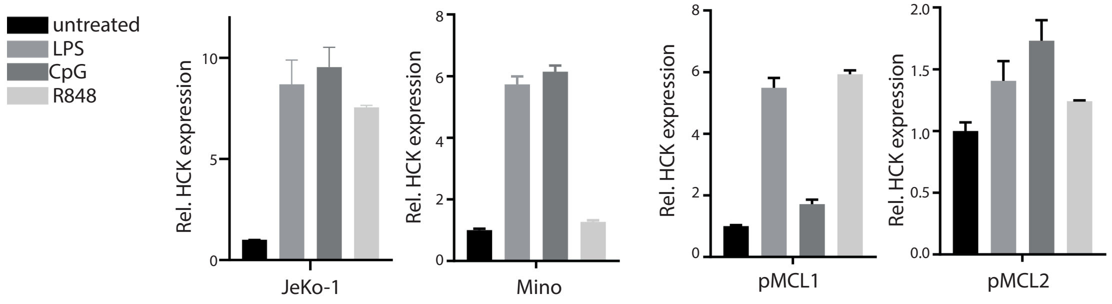

HCK mRNA expression in primary MCL material and cell lines stimulated for 3 hours with LPS, CpG or R848 determined by RT-qPCR. RPLP0 was used as an input control. Different y-axis scales were used in order to visualize the various stimuli in each cell type. Data were normalized to the value of untreated cells.
