# Supplementary material for: Identification of the SRC-family tyrosine kinase HCK as a therapeutic target in mantle cell lymphoma
Source: Leukemia. 2020 Jun 26;35(3):881–6. doi: 10.1038/s41375-020-0934-6 (PMC7932922; doi:10.1038/s41375-020-0934-6)
Supplement: Supplementary file 2 — Supplemental Figure 4 [file 41375_2020_934_MOESM2_ESM.pdf]

Supplementary Figure 4

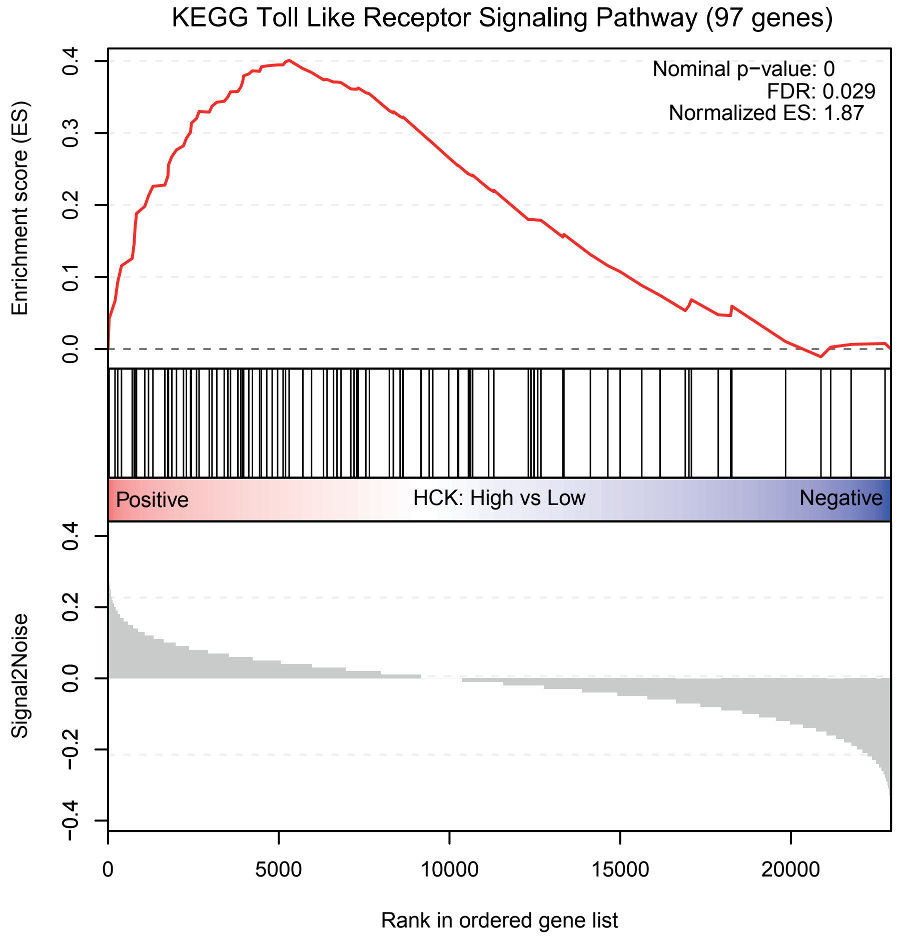

Enrichment plot of HCK High (n=41) versus Low (n=81) patients on the curated KEGG geneset Toll Like Receptor Signaling Pathway. FDR = False Discovery Rate, ES = Enrichment Score
