# Supplementary material for: Identification of the SRC-family tyrosine kinase HCK as a therapeutic target in mantle cell lymphoma
Source: Leukemia. 2020 Jun 26;35(3):881–6. doi: 10.1038/s41375-020-0934-6 (PMC7932922; doi:10.1038/s41375-020-0934-6)
Supplement: Supplementary file 3 — Supplemental Figure 5 [file 41375_2020_934_MOESM3_ESM.pdf]

## Supplementary Figure 5

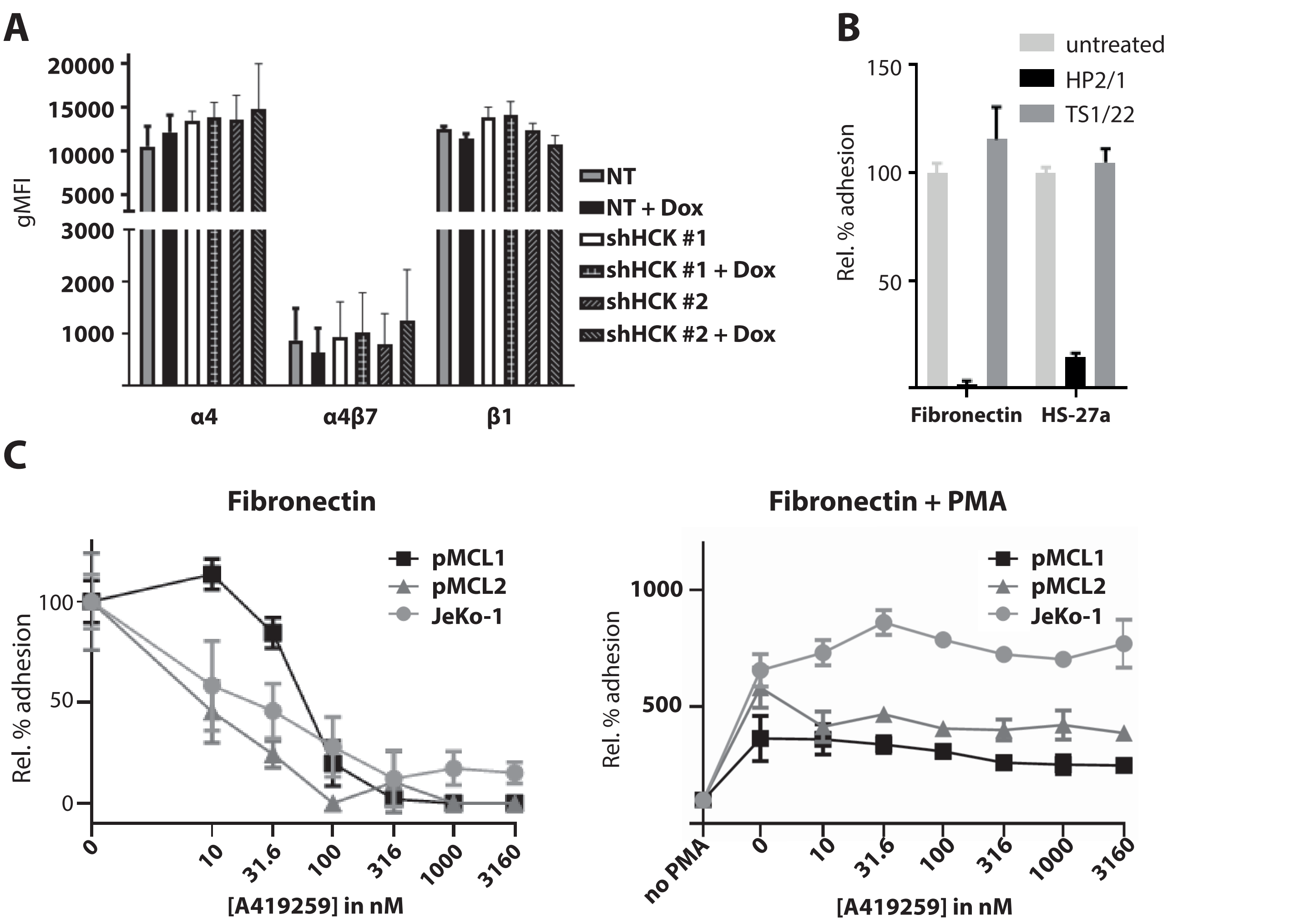


(A) Expression of various integrin subtypes after HCK knockdown in JeKo-1 cels transduced with pLKO-TET-puro plasmids encoding two shRNA's targeting HCK, as determined by flow cytometric analysis after 4 days of doxycycline treatment. Data are presented as mean +/- S.E.M of three independent experiments. (B) Integrin-mediated adhesion of JeKo-1 cells after treatment with blocking antibodies against integrin-α4 (HP2/1) or integrin-αL (TS1/22). Cells were treated for 30 minutes at 4˚C and then allowed to adhere to fibronectin-coated plates or a monolayer of HS-27a-GFP stromal cells for 30 minutes. Non-adherent cells were removed by extensive washing, and adherent cells were quantified. Percentages of adherent cells were normalised to the untreated condition. Data are presented as mean +/- S.D. (C) Integrin-mediated adhesion of primary MCL or JeKo-1 cells after treatment with A419259 with or without PMA. Cells were treated for 30 minutes with various concentrations of A419259 in the absence (left panel) or presence (right panel) of PMA and allowed to adhere to fibronectin-coated plates for 30 minutes. Non-adherent cells were removed by extensive washing, and adherent cells were quantified. Percentages of adherent cells were normalized to the untreated condition without PMA.
