# Supplementary material for: Identification of the SRC-family tyrosine kinase HCK as a therapeutic target in mantle cell lymphoma
Source: Leukemia. 2020 Jun 26;35(3):881–6. doi: 10.1038/s41375-020-0934-6 (PMC7932922; doi:10.1038/s41375-020-0934-6)
Supplement: Supplementary file 5 — Supplemental Materials and Methods [file 41375_2020_934_MOESM5_ESM.docx]

**Supplementary Methods and Figures**

**Cell culture and treatments**

The MCL cell lines JeKo-1, Granta-519, Maver-1, Z-138, Rec-1 and Mino; the DLBCL cell lines OCI-Ly1, OCI-Ly7; and the WM cell lines BCWM.1 and MWCL-1 (both kindly provided by Dr. Zachary Hunter) were cultured in IMDM medium supplemented with 10 % fetal calf serum. The DLBCL cell lines Riva and U2932 were cultured in RPMI-1640 medium supplemented with 10 % fetal calf serum, HBL-1 and TMD8 supplemented with 20 % fetal calf serum and OCI-Ly10 supplemented with 20 % human serum (Sigma-Aldrich). The stromal cell line HS-27a was cultured in DMEM supplemented with 10 % fetal calf serum. All cells were cultured with supplement of 2 mM L-glutamine, 100 units/mL penicillin and 100 µg/mL streptomycin. Cell lines were routinely authenticated by STR profiling (Promega, Madison, Wisconsin, USA) and monitored for contaminations with mycoplasmas. Peripheral blood derived MCL cells were obtained after routine diagnostics or follow-up procedures at the department of Hematology of the Amsterdam University Medical Centers, location AMC, the Netherlands, and were purified using Ficoll and B cell isolation kit (Milteny Biotec.) Purified MCL samples were sorted on a BD-FACS-Aria IIu to obtain CD5^+^/CD19^+^ cells and were cultured in IMDM supplemented with 20 % fetal calf serum, 2 mM L-glutamine, 100 units/ml penicillin and 100 µg/ml streptomycin. This study was approved by the AMC Medical Committee on Human Experimentation. Informed consent was obtained in accordance with the revised Declaration of Helsinki 2008.

Naïve B-cells (CD19^+^/IgD^+^/CD38^-^), germinal center B-cells ( (CD19^+^/IgD^-^/CD38^+^), memory B-cells (CD19^+^/IgD^-^/CD38^-^) and plasmablasts (CD19^+^/IgD^-^/Cd38^hi^) were isolated from tonsil and sorted on a BD FACS-Aria IIu (BD Biosciences).

For inducible shRNA-mediated knockdown, cells were incubated with 3 µg/ml doxycycline for 3-7 days as indicated. MCL cell were stimulated with 1 µg/ml LPS (Sigma-Aldrich), 0.05 µM CPG (Invivogen) or 1 µg/ml R848 (Invivogen) for 3 hours (RNA) or 48 hours (immunoblot).

**Transfection and transduction**

shRNA’s against HCK #1 (CCAGGTCGGAGGCAATACATT), #2 (CAGGGAGATACCGTGAAACAT) and against MYD88 #1 (CCTGTCTCTGTTCTTGAACGT) and #2 (GCAGAGCAAGGAATGTGACTT) were inserted in Tet-pLKO-puro (Gift from Dmitri Wiederschain, Addgene #21915) as previously described ^1^. Lentiviral particles were generated by cotransfection of pMD2.G (Gift from Didier Trono, Addgene #12259), pPAX2 (Gift from Didier Trono, Addgene #12260) and the lentiviral shRNA encoding vector in a 1:2:4 ratio with Genius DNA transfection reagent (Westburg) according to the manufacturer’s instructions. Forty-eight hours after transfection, lentiviral particles were harvested and MCL cells were spinofected for 60 minutes at 1800 x g at 33° C in the presence of 8 µg/ml polybrene (Sigma-Aldrich). Three days after transduction the cells were selected with increasing concentrations of puromycin (Invivogen) for 10-14 days (JeKo-1) or 18-21 days (Granta-519). Similarly, HS-27a was transduced with pLKO-GFP, with the exception that transduced GFP positive cells were sorted on a Sony SH800s cell sorter.

**Immunoblotting**

Protein lysates (whole-cell extracts in RIPA-buffer) were separated on Bolt ™ 4-12% Bis-Tris Plus gels (Invitrogen) and subsequently blotted to a PVDF-membrane. The antibodies used were: mouse anti-β-actin (AC-15, Sigma-Aldrich), rabbit anti-HCK (E1I7F, Cell Signaling Technology), rabbit anti-MYD88 (D80F5, Cell Signaling Technology), rabbit anti-LYN (44, Santa Cruz Biotechnology) and rabbit anti-LCK (2102, Santa Cruz Biotechnology). Primary antibodies were detected with anti-mouse-HRP or anti-rabbit-HRP (both DAKO), followed by detection using Pierce™ ECL Western Blotting Substrate (Thermo Scientific).

**RT-qPCR**

Total RNA was isolated using TRI-reagent (Sigma-Aldrich), according to the manufacturer’s protocol. RNA was converted to cDNA using oligo-DT. PCRs were performed using Sensifast (Bioline) on a CFX-384 RT-PCR detection system (Bio-Rad) and obtained Ct values were normalized to those of the input control *RPLP0*. Primers used were: *HCK* forward (TGGCAGTGAAGACGATGAAG), *HCK* reverse (GTAGATGGGCTCCTTGGTGA), *RPLP0* forward (GCTTCCTGGAGGGTGTCCGC) and *RPLP0* reverse (TCCGTCTCCACAGACAAGGCCA)

**Adhesion assay**

The adhesion assays were essentially performed as previously described ^2,3^. In detail, adhesion assays were performed in triplicate on Microlon® high binding plates (Greiner) coated overnight with PBS containing 10 µg/ml fibronectin (Sigma-Aldrich) or 4 % BSA (Sigma-Aldrich) at 4° C overnight or with 1 mg/ml poly-L-lysine (PLL, Sigma-Aldrich) for 15 minutes at 37° C. On the day of the experiment plates were blocked for 1h at 37° C with IMDM/4 % BSA. In the case of adhesion to the stromal cell line HS-27a-GFP, 15.000 cells were seeded the day prior to the experiment in order to form a confluent cell monolayer. As indicated, cells were treated for 30 minutes with A419259 at 37° C or Hp2/1 (Millipore) and TS1/22 (Invitrogen) at 4° C. Per well 1,5*10^5 JeKo-1 or 5*10^5 primary MCL cells were plated and incubated with or without PMA (50 ng/ml) at 37° C for 30 minutes. After extensive washing of the plate with IMDM/0,5 % BSA to remove non-adherent cells, the cells were fixed for 10 minutes with 10 % glutaraldehyde/PBS (Millipore) and subsequently stained with 0,4 % crystalviolet/20 % ethanol for 45 minutes. After thorough washing with ddH_2_O, ethanol was added for 30 minutes to elute the dye and quantified by measuring the absorbance at 570 nm on a spectrophotometer (Clariostar, BMG Labtech). In the case of adhesion to stromal cell line HS-27a-GFP, cells were not fixed but trypsinised and subsequently quantified by flow cytometric analysis (FACS CantoII, BD Biosciences). When analyzing adhesion to fibronectin, absorbance due to nonspecific adhesion, as determined in wells coated with 4% BSA, was subtracted. Maximal adhesion (100%) was determined by applying the cells to wells coated with PLL, without washing the wells before fixation or, in the case of adhesion to HS-27a, by counting the number of input cells.

**Annexin-V staining**

Annexin-V staining was essentially performed as previously described ^4^. Briefly, cells were stained with Annexin-V-FITC in Annexin binding buffer followed by staining with To-Pro-3 and analysis on a FACS CantoII. Apoptotic cells were defined as Annexin-V^+^. Typically JeKo-1 cells showed approximately 7 % Annexin-V^+^ cells at baseline and Granta-519 13 % respectively.

**BrdU cell-cycle analysis**

For cell-cycle analysis, cells were incubated for 1 hour with 20 µM BrdU (Sigma-Aldrich), washed once with PBS/0,1 % BSA and subsequently fixed in ice-cold 75 % ethanol/PBS. After washing, the cells were incubated with 0,4 mg/ml pepsin in 0.2 mM HCl for 30 minutes at room temperature and subsequently with 2 N HCl for 25 minutes at 37° C. Cells were washed once with PBT (PBS/0,05 % Tween-20) and once with PBT_B_ (PBT/2 % BSA) and stained for 30 minutes with anti-BrdU-FITC (clone B44, BD Biosciences) in PBT_B_. After washing with PBT and PBT_B_, cells were treated with 500 µg/ml RNAse A (Bioke) and stained with 0,1 µM To-Pro-3 (Invitrogen) in PBS/1 % BSA/0,05 % NaN_3_ for 15 minutes at 37° C followed by analysis on a FACS Canto II (BD Biosciences).

**Flow Cytometric analysis**

10^5^ cells were stained with anti-α_4_-integrin ( HP2/1, Millipore), anti-α_4_β_7_-integrin ^5^, anti-β_1_-integrin (4B4, Coulter Immunology), or control mouse anti-IgG_1_, followed by staining with PE-conjugated anti-mouse IgG_1_. Cell surface staining was analyzed on a FACSCantoII (BD Biosciences).

**Microarray analysis**

The following gene expression data, publically available and deposited in the NIH Gene Expression Omnibus database, were analyzed using the R2 Genomics Analysis and Visualization Platform (r2.amc.nl): GSE12366 (healthy B-cells, excluding undefined B-cells), GSE10846 (DLBCL, excluding unclassified subtypes), GSE93921 (MCL) , GSE9656 (WM), GSE39671 (CLL) and GSE2658 (MM). The overall survival of the tertile of MCL patients (GSE93921) with the highest HCK expression (HCK high) was compared to the rest (HCK low). All datasets were obtained with the Affymetrix U133p2 array and normalized using the MAS 5.0 algorithm to allow comparison between datasets.

**Gene Set Enrichment Analysis**

Gene Set Enrichment Analysis (GSEA) was performed using the GSEA application (version 4.0.3) on microarray data comparing the HCK high versus HCK low MCL patients on the curated KEGG pathway gene sets. GSEA plots were redrawn using the replotGSEA function from the Rtoolbox package (<https://github.com/PeeperLab/Rtoolbox>).

**References**

1. Wiederschain D, Wee S, Chen L, et al. Single-vector inducible lentiviral RNAi system for oncology target validation. *Cell Cycle.* 2009;8(3):498-504.

2. Spaargaren M, Beuling EA, Rurup ML, et al. The B cell antigen receptor controls integrin activity through Btk and PLCgamma2. *J Exp Med.* 2003;198(10):1539-1550.

3. de Rooij MF, Kuil A, Geest CR, et al. The clinically active BTK inhibitor PCI-32765 targets B-cell receptor- and chemokine-controlled adhesion and migration in chronic lymphocytic leukemia. *Blood.* 2012;119(11):2590-2594.

4. Koopman G, Reutelingsperger CP, Kuijten GA, Keehnen RM, Pals ST, van Oers MH. Annexin V for flow cytometric detection of phosphatidylserine expression on B cells undergoing apoptosis. *Blood.* 1994;84(5):1415-1420.

5. Lazarovits AI, Moscicki RA, Kurnick JT, et al. Lymphocyte activation antigens. I. A monoclonal antibody, anti-Act I, defines a new late lymphocyte activation antigen. *J Immunol.* 1984;133(4):1857-1862.
